# Transferrin receptor 1 upregulation in primary tumor and downregulation in benign kidney is associated with progression and mortality in renal cell carcinoma patients

**DOI:** 10.18632/oncotarget.22323

**Published:** 2017-11-06

**Authors:** Christopher J. Greene, Kristopher Attwood, Nitika J. Sharma, Kenneth W. Gross, Gary J. Smith, Bo Xu, Eric C. Kauffman

**Affiliations:** ^1^ Department of Urology, Roswell Park Cancer Institute, Buffalo, NY 14263, USA; ^2^ Department of Biostatistics and Bioinformatics, Roswell Park Cancer Institute, Buffalo, NY 14263, USA; ^3^ Department of Molecular and Cellular Biology, Roswell Park Cancer Institute, Buffalo, NY 14263, USA; ^4^ Department of Pathology, Roswell Park Cancer Institute, Buffalo, NY 14263, USA; ^5^ Department of Cancer Genetics, Roswell Park Cancer Institute, Buffalo, NY 14263, USA; ^6^ Jacobs School of Medicine and Biomedical Sciences, State University of New York at Buffalo, Buffalo, NY 14214, USA

**Keywords:** transferrin receptor, iron, renal cell carcinoma, clear cell, immunohistochemistry

## Abstract

The central dysregulated pathway of clear cell (cc) renal cell carcinoma (RCC), the von Hippel Lindau/hypoxia inducible factor-α axis, is a key regulator of intracellular iron levels, however the role of iron uptake in human RCC tumorigenesis and progression remains unknown. We conducted a thorough, large-scale investigation of the expression and prognostic significance of the primary iron uptake protein, transferrin receptor 1 (TfR1/CD71/TFRC), in RCC patients. TfR1 immunohistochemistry was performed in over 1500 cores from 574 renal cell tumor patient tissues (primary tumors, matched benign kidneys, metastases) and non-neoplastic tissues from 36 different body sites. TfR1 levels in RCC tumors, particularly ccRCC, were significantly associated with adverse clinical prognostic features (anemia, lower body mass index, smoking), worse tumor pathology (size, stage, grade, multifocality, sarcomatoid dedifferentiation) and worse survival outcomes, including after adjustments for tumor pathology. Highest TfR1 tissue levels in the non-gravid body were detected in benign renal tubule epithelium. Opposite to TfR1 changes in the primary tumor, TfR1 levels in benign kidney dropped during tumor progression and were inversely associated with worse survival outcomes, independent of tumor pathology. Quantitative measurement of TfR1 subcellular localization in cell lines demonstrated mixed cytoplasmic and membranous expression with increased TfR1 in clusters in ccRCC versus benign renal cell lines. Results of this study support an important role for TfR1 in RCC progression and identify TfR1 as a novel RCC biomarker and therapeutic target.

## INTRODUCTION

More than 60,000 new cases of kidney cancer will be diagnosed this year in the U.S., with over 90% being renal cell carcinoma (RCC) [1, 2]. RCC is comprised of different subtypes, each with a distinct histology, genetic mutational profile and clinical behavior, and includes clear cell RCC (ccRCC, 75%), papillary RCC (pRCC, 15%), chromophobe RCC (chRCC, 5%) and less common subtypes [3-6]. Risk factors include male gender, tobacco, hypertension and obesity, but may vary with RCC subtype [2, 7, 8]. Despite recent clinical advances, RCC diagnoses are increasing annually by 2-3% without reduction in mortality, and metastatic relapse after nephrectomy for localized disease remains a frequent event [9-11]. Current systemic therapies approved for advanced disease achieve only modest survival benefits [12], and no prognostic molecular biomarkers are presently available to guide RCC patient management [13]. Hence there is a critical need for improved prevention and treatment strategies.

Iron is the most abundant transition metal in the human body and a potent catalyst of intracellular oxidative stress, a process heavily implicated in RCC tumorigenesis [6, 14-17]. Through the Fenton reaction, iron reacts with hydrogen peroxide to produce hydroxyl radicals that act as potent, non-selective oxidizing agents of cellular macromolecules including protein, lipid, carbohydrate and nucleic acids [18, 19]. Oxidative DNA damage can induce gene mutations, chromosomal remodeling and epigenetic instability that may contribute to carcinogenesis [20]. Furthermore, iron drives cell division, a hallmark of tumorigenesis, as the essential cofactor for the rate-limiting step of DNA synthesis, and is also critical for other processes implicated in carcinogenesis, which include chromatin remodeling by the iron-dependent oxidase superfamily; DNA repair by iron-dependent enzymes such as p53R2/RRM2B; cellular/mitochondrial respiration involving iron-sulphur cluster proteins; and cell cycle progression through indirect effects on p53, Rb, p21 and p27 [21-24].

The kidney has a unique role in iron physiology, providing the main body source of the hormone, erythropoietin, which mobilizes iron stores via hemoglobin synthesis [25]. Animal studies provide compelling support that iron might play an under-appreciated role in RCC tumorigenesis. Repeated systemic administration of iron to rodents causes iron deposition with oxidative tissue injury in renal tubule epithelium, the presumed site of human RCC tumorigenesis, followed by renal tumorigenesis that mimics human ccRCC in histology, male gender predominance, and metastatic affinity for the lungs and lymph nodes [26-30]. In humans, increased RCC rates are reported among workers in iron/steel industries; and among patients with anemias requiring chronic transfusion, a primary cause of systemic iron overload [31, 32]. Iron is also a prominent component of tobacco, the most well-established RCC carcinogen [33]. Despite these compelling observations, a role for altered iron metabolism in human RCC has undergone scarce investigation [34, 35].

Regulation of intracellular iron metabolism is governed by a distinct set of proteins [36]. The primary protein for intracellular iron uptake is transferrin receptor 1 (TfR1/CD71/TFRC), which is constitutively endocytosed from the cell membrane upon binding iron with its serum carrier protein, transferrin (Tf). The more recently discovered transferrin receptor 2 (TfR2) has restricted tissue expression (liver, duodenum, erythrocytes) and a minor role in iron uptake [37, 38]. Intracellular iron transport, storage and export are governed by divalent metal transporter 1 (SLC11A2/DMT1), ferritin (heavy chain, FTH1, and light chain, FTL), ferroportin (SLC40A1/FPN1) and hepcidin (HAMP). During iron deficiency, iron regulatory proteins (ACO1/IRP1, IREB2/IRP2) increase iron levels by binding to a small subset of mRNA transcripts that harbor an iron responsive element (IRE) to modulate their translation. IRE motifs are restricted to transcripts whose products are critical to iron metabolism, such as TfR1, DMT1, FTH1, FTL and FPN1 [39].

Additional regulation of iron metabolism is provided by the centrally dysregulated pathway of ccRCC, the von Hippel Lindau (VHL)/proline hydroxylase (PHD)/hypoxia inducible factor-α (HIF-α) axis, which governs the cell response to intracellular oxygen and iron levels [3]. The *VHL* gene is mutated in 55-75% of ccRCC patient tumors [4, 40, 41], and an additional subset of ccRCC tumors harbor *VHL* promoter hypermethylation [42], resulting in VHL functional loss in approximately 90% of ccRCC tumors [43, 44]. VHL loss causes HIF-α transcription factor accumulation and transcriptional activation of HIF-α target genes that include the TfR1 gene, *TFRC* [45-47]. HIF-2α is believed to be the critical VHL target driving ccRCC carcinogenesis, since HIF-2α but not HIF-1α is overexpressed in potentially all VHL-null ccRCC patient tumors [44]; overexpression of HIF-2α but not HIF-1α is sufficient to restore ccRCC cell line tumorigenicity suppressed by VHL overexpression [48, 49]; and higher RCC tumor levels of HIF-2α portend a worse patient prognosis [50]. Intriguingly, an IRE binding site for IRP1 has been identified in the 5’ UTR of the HIF-2α transcript that increases HIF-2α translation in the presence of iron [51]. However, the role of iron uptake in modulating HIF-2α expression in RCC patient tumors remains unknown.

The current study provides a thorough, large-scale investigation into the level and prognostic significance of TfR1 expression in renal cell tumor patients. Our findings reveal significant associations of TfR1 expression in RCC primary tumors with disease progression and patient mortality, particularly for ccRCC patients. Furthermore, we discover that the highest non-gravid body levels of TfR1 protein are in benign kidney, underscoring a unique role for this protein in renal physiology/disease; and that, opposite to changes in the primary tumor, reductions in benign kidney TfR1 are associated with more aggressive RCC tumors. Together, these data support a complex, tissue-specific role for TfR1 in RCC progression and identify this protein as a novel potential RCC biomarker and therapeutic target.

## RESULTS

### TfR1 expression in normal human tissues

**Figure 1:**
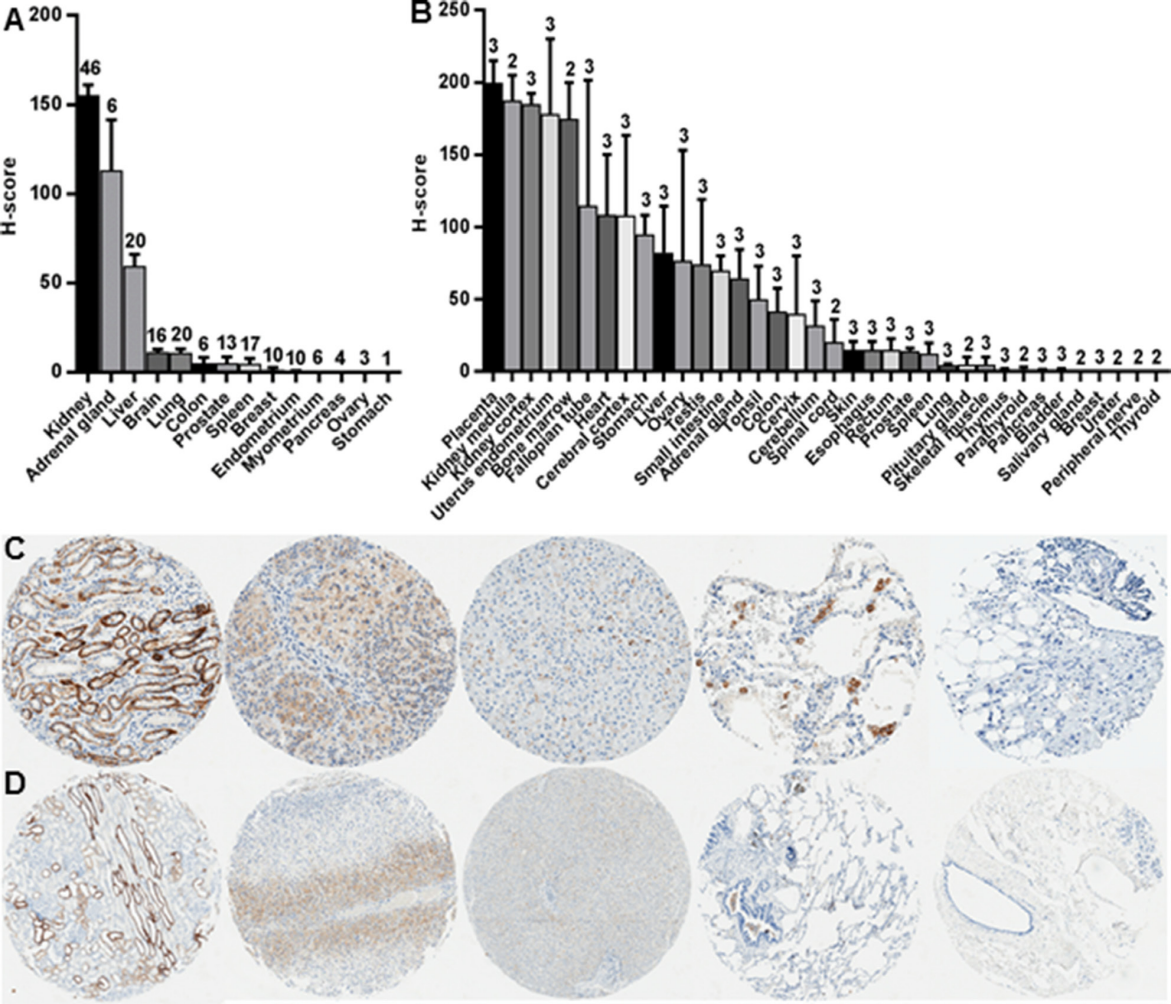
TfR1 levels in normal tissues of various body sites IHC was performed for TfR1 in normal (non-neoplastic) tissues using two different TMA sources: **(A)** RCC patient TMAs which included 14 different normal tissue types as internal staining controls; and **(B)** a normal tissue TMA surveying 36 different body-wide tissue types. Representative tissue cores are shown for (left to right): kidney cortex, adrenal gland, liver, lung and breast from the **(C)** 14-tissue TMAs and **(D)** 36-tissue normal TMA. The number of cores evaluated per tissue type is labeled above each error bar.

TfR1 protein levels were evaluated by IHC in non-neoplastic human tissue types from two sources: 1) RCC patient tissue microarrays (TMA(s)) which included 14 different normal tissue types as internal staining controls, and 2) a normal tissue TMA surveying 36 different body tissue types (Figure 1). RCC patient TMAs immunostain detected little or no TfR1 protein expression (<5% percentage of tissue positivity, PTP) in most normal body tissues, with the exception of kidney, adrenal gland and liver, which had moderate to high levels. Highest TfR1 levels were detected in the kidney (Figure 1A) and localized predominantly to the basal membrane and cytoplasm of renal tubule epithelium, without expression in adjacent stroma (Figure 1C). Distal tubule staining intensity was typically high (3+), while proximal tubule staining intensity was typically moderate (2+). Similar results were obtained using the commercial normal tissue TMA, in which only placenta had greater TfR1 expression than kidney among the 36 tissue types tested (Figure 1B, 1D). There was no appreciable difference in TfR1 expression between the renal cortex and renal medulla.

### Renal cell tumor patient features

Among 287 renal cell tumor patients represented on the TMAs, 574 tissues (268 primary tumors, 232 matching normal kidneys, 74 metastases) had adequate TfR1 staining for evaluation (mean/median 2.7/3.0 cores per specimen). Clinicopathologic features of patients and tissues are summarized in Table 1. Most patients had ccRCC, and 11 patients had benign renal tumors (oncocytoma) without evidence of RCC. RCC risk factors of male gender, smoking history, hypertension and obesity (body mass index, BMI, >30 kg/m^2^) were common. More than one third of patients were anemic and 13 patients were known to be on iron supplementation. Of the 74 metastasis tissues, most were either ccRCC or an unknown RCC subtype, and only 7 metastasis tissues were from non-ccRCC tumors.

**Table 1:** Renal cell tumor patient and tissue characteristics

	Patients, n (%)	Tissues, n (%)		
		Primary Tumor	Benign Kidney	Metastasis
Total number	287	268	232	74
Age, years				
<= 60	155 (54.0)	144 (53.7)	119 (51.3)	52 (70.3)
> 60	132 (46.0)	124 (46.3)	113 (48.7)	22 (29.7)
Gender				
Male	179 (62.4)	166 (61.9)	147 (63.4)	46 (62.2)
Female	108 (37.6)	102 (38.1)	85 (36.6)	28 (37.8)
Race				
White	268 (93.4)	252 (94.0)	218 (94.0)	71 (95.9)
Black	19 (6.6)	16 (6.0)	14 (6.0)	3 (4.1)
Body Mass Index, kg/m^2^				
< 30	109 (49.8)	120 (51.3)	101 (50.8)	16 (66.7)
>= 30	110 (50.2)	114 (48.7)	98 (49.2)	8 (33.3)
Smoking History				
Never	122 (43.7)	118 (45.0)	103 (45.6)	21 (29.2)
Any	157 (56.3)	144 (55.0)	123 (54.4)	51 (70.8)
Smoking Pack-Years				
0	122 (56.0)	118 (52.4)	103 (54.2)	21 (58.3)
<= 30	56 (25.7)	64 (28.4)	49 (25.8)	10 (27.8)
> 30	40 (18.3)	43 (19.1)	38 (20.0)	5 (13.9)
Iron Supplement Medication				
No	199 (94.8)	213 (94.2)	180 (94.2)	22 (88.0)
Yes	11 (5.2)	13 (5.8)	11 (5.8)	3 (12.0)
Anemia				
No	91 (64.1)	92 (61.3)	82 (62.1)	2 (14.3)
Yes	51 (35.9)	58 (38.7)	50 (37.9)	12 (85.7)
Iron-Deficient Anemia				
No	112 (90.3)	116 (89.9)	103 (90.4)	4 (50.0)
Yes	12 (9.7)	13 (10.1)	11 (9.6)	4 (50.0)
Hemoglobin level, g/dL				
< 13.3	63 (44.4)	69 (46.0)	60 (45.5)	11 (78.6)
>= 13.3	79 (55.6)	81 (54.0)	72 (54.5)	3 (21.4)
Hypertension				
No	64 (32.5)	71 (33.3)	61 (33.9)	11 (45.8)
Yes	133 (67.5)	142 (66.7)	119 (66.1)	13 (54.2)
Histologic Subtype				
ccRCC	195 (67.9)	203 (75.7)	176 (75.9)	34 (45.9)
pRCC	26 (9.1)	25 (9.3)	22 (9.5)	6 (8.1)
chRCC	13 (4.5)	12 (4.5)	11 (4.7)	0 (0)
Other RCC	3 (1.0)	3 (1.1)	1 (0.4)	1 (1.4)
Unspecified RCC	39 (13.6)	14 (5.2)	12 (5.2)	33 (44.6)
Oncocytoma	11 (3.8)	11 (4.1)	10 (4.3)	0 (0)
Tumor Grade				
I	14 (5.4)	13 (5.2)	12 (5.5)	1 (1.6)
II	127 (49.0)	116 (46.2)	104 (47.5)	19 (30.6)
III	72 (27.8)	68 (27.1)	57 (26.0)	20 (32.3)
IV	46 (17.8)	54 (21.5)	46 (21.0)	22 (35.5)
Tumor Stage				
pT1	131 (47.5)	131 (51.0)	114 (51.4)	5 (6.8)
pT2	43 (15.6)	44 (17.1)	38 (17.1)	8 (10.8)
pT3/4	70 (25.4)	81 (31.5)	69 (31.1)	30 (40.5)
pTx	32 (11.6)	1 (0.4)	1 (0.5)	31 (41.9)
Tumor Size, cm				
<= 4	97 (35.7)	94 (35.7)	84 (37.0)	7 (11.5)
> 4 and </= 7	84 (30.9)	76 (28.9)	64 (28.2)	20 (32.8)
> 7 and </= 10	49 (18.0)	50 (19.0)	45 (19.8)	18 (29.5)
> 10	42 (15.4)	43 (16.3)	34 (15.0)	16 (26.2)
Tumor Focality				
Unifocal	168 (88.0)	178 (87.3)	155 (88.1)	17 (81.0)
Multifocal	23 (12.0)	26 (12.7)	21 (11.9)	4 (19.0)
Tumor Presence of Sarcomatoid				
No	218 (91.6)	230 (90.2)	196 (89.9)	18 (66.7)
Yes	20 (8.4)	25 (9.8)	22 (10.1)	9 (33.3)
Tumor Sarcomatoid Percentage				
0	220 (93.2)	232 (91.7)	198 (91.7)	20 (74.1)
<= 50%	8 (3.4)	10 (4.0)	8 (3.7)	3 (11.1)
> 50%	8 (3.4)	11 (4.3)	10 (4.6)	4 (14.8)

### TfR1 expression in benign kidney, primary tumor and metastasis tissues of renal cell tumor patients

**Figure 2:**
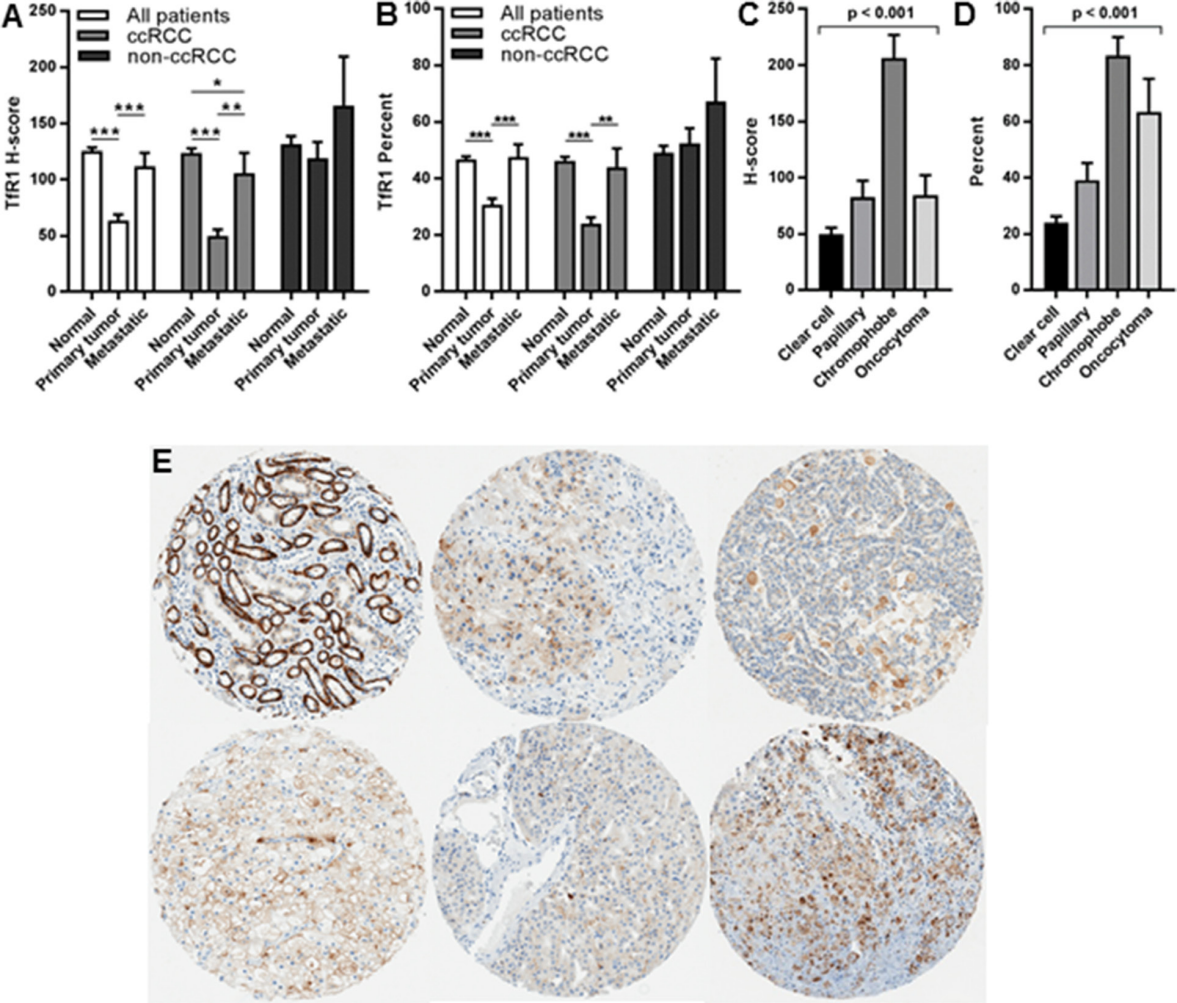
TfR1 levels in normal kidney, primary tumors and metastases of renal cell tumor patients IHC was performed for TfR1 using renal cell tumor patient TMAs. TfR1 levels were compared among normal (non-neoplastic) kidney, primary tumors and metastatic lesions, based on **(A)** mean H-score and **(B)** mean PTP. TfR1 levels were compared among different renal cell primary tumor subtypes based on **(C)** mean H-score and **(D)** mean PTP. **(E)** Representative images of TfR1 staining from different kidney tissue types; top row: normal kidney (left), ccRCC (middle), pRCC (right); bottom row: chRCC (left), oncocytoma (middle), metastatic ccRCC (right). ^*^, p<0.05; ^**^, p<0.01; ^***^, p<0.001.

TfR1 protein was detected by IHC at high levels in normal kidney tissue from renal cell tumor patients, similar to levels in non-matching normal kidney tissue controls. Expression was maintained in primary tumors, although at significantly lower levels compared to matching normal renal epithelium (Figure 2A, 2B). The exception was chRCC, which had slightly higher expression of TfR1 (mean PTP= 83%, mean H-score= 206) than matching benign kidney. Lowest TfR1 tumor expression was detected in ccRCC tumors (mean PTP= 24%, mean H-score= 50). TfR1 levels in other renal tumor subtypes, including pRCC or benign oncocytoma, were intermediate between TfR1 levels of ccRCC and chRCC (Figure 2C, 2D, and 2E). In subset analyses limited to matched primary tumor and benign kidney tissues, tumor TfR1 levels remained significantly (p<0.001) lower than normal kidney TfR1 levels; and there was a non-significant trend towards a negative correlation between TfR1 levels in tumor and normal kidney from the same patient (Supplementary Table 1).

Mean TfR1 levels in metastases were significantly higher than in primary tumors from metastatic and non-metastatic patients (Figure 2A, 2B), reaching approximately 2-fold higher levels among ccRCC patients. However, in subset analyses limited to only metastatic patients, TfR1 levels in metastases and primary tumors were nearly identical; and there was a statistically significant correlation of moderate strength between TfR1 levels in primary tumor and metastatic tissue from the same patient (Supplementary Table 2).

TfR1 subcellular localization in tumors and metastases was generally both membranous and cytoplasmic and less frequently nuclear, perinuclear, or isolated membranous without cytoplasmic involvement.

### Association of renal cell primary tumor TfR1 expression with preoperative clinical features

Primary tumor TfR1 protein levels (PTP and H-score) were tested for association with preoperative clinical features of renal cell tumor patients, including known diagnostic and prognostic risk factors (Table 2). Primary tumor TfR1 expression was significantly increased in patients with metastatic stage or certain adverse prognostic risk factors, including a lower BMI and a lower hemoglobin or history of anemia. An iron-deficient type of anemia (and hence the use of iron supplementation) was associated with the highest tumor TfR1 levels among clinical features examined. These findings were confirmed among RCC patients after exclusion of benign oncocytoma patients (data not shown).

**Table 2:** Association between primary tumor TfR1 level and renal cell tumor patient clinical features

	All Patients		ccRCC Patients		Non-ccRCC patients	
	TfR1 PTP	TfR1 H-score	TfR1 PTP	TfR1 H-score	TfR1 PTP	TfR1 H-score
	(mean/SE)	(mean/SE)	(mean/SE)	(mean/SE)	(mean/SE)	(mean/SE)
Age, years						
<= 60	33.2/3.1	70.5/7.6	26.4/3.3	54.8/8.1	60.5/7.3	136.3/19.5
> 60	27.8/2.9	55.9/6.8	21.0/3.0	44.1/7.5	41.3/7.6	95.7/21.1
P-value	0.44	0.48	0.52	0.69	0.095	0.13
Gender						
Male	32.7/2.8	67.5/6.7	26.6/3.0	55.4/7.4	51.5/7.0	121.8/18.6
Female	27.5/3.4	57.6/8.1	19.7/3.4	41.2/8.4	54.0/9.1	113.9/23.7
P-value	0.34	0.37	0.34	0.31	0.87	0.88
Race						
White	30.9/2.2	64.4/5.4	24.6/2.3	51.3/5.7	55.3/6.1	128/16.4
Black	30/8.6	56.6/17.4	4.22/3.5	5.11/3.7	38.4/11.8	76.7/26.9
P-value	0.40	0.40	0.22	0.22	0.33	0.29
Body Mass Index, kg/m^2^						
< 30	34/3.3	72.3/8.3	30/3.7	66.8/9.7	45.7/8.0	97.6/20.3
>= 30	25.5/3.2	50.2/7.3	16.5/2.9	29.8/6.2	59.3/9.0	144.8/25.0/17
P-value	0.052	**0.031**	**0.011**	**0.006**	0.19	0.18
Smoking History
Never	28.9/3.2	58/7.6	22/2.3	44.3/8.2	54.7/8.1	121.7/21.2
Any	33/3.0	70/7.2	26.6/3.2	56.7/7.9	50.2/7.9	116.5/20.3
P-value	0.13	0.076	0.071	0.053	0.58	0.85
Pack Years						
0	28.9/3.2	58/7.6	22/2.3	44.3/8.2	54.7/7.6	121.7/21.2
<= 30	30.1/4.6	64.7/11.1	26/4.9	58.4/12.5	42.0/13.0	91/30.7
> 30	33.3/5.0	70.1/12.7	28.3/5.4	59.7/13.6	55.0/11.6	121.4/36.5
P-value	0.34	0.21	0.12	0.082	0.54	0.42
Iron Supplement Medication						
No	28.3/2.4	57.6/5.7	21.2/2.4	43.7/6.0	49.9/6.4	113.7/17.2
Yes	51.8/10.0	116/25.5	48.3/12.9	103.5/32.2	62.5/9.2	170.8/17.5
P-value	**0.038**	**0.022**	**0.048**	**0.005**	0.57	0.41
Anemia						
No	23.7/3.1	41.8/6.0	14/2.4	24.8/4.8	43.8/8.0	89.5/19.5
Yes	44.1/5.3	101/13.9	37.6/6.2	86.7/16.5	65.4/9.7	162.1/27.0
P-value	**0.003**	**0.001**	**0.008**	**0.008**	0.088	**0.03**
Iron-Deficient Anemia						
No	27.9/3.0	53.5/6.7	17.9/2.9	35.1/6.9	47.6/6.7	102.3/16.8
Yes	73.5/10.8	184.1/30.6	69.7/12.5	175.0/35.3	94.2/2.5	234.2/42.5
P-value	**<.001**	**<.001**	**0.001**	**0.001**	0.078	0.052
Hemoglobin, g/dL						
< 13.3	41.1/4.8	92.6/12.3	34.8/5.5	79.6/14.5	57.4/9.9	140.5/27.3
>= 13.3	23.6/3.3	40.8/6.2	13.2/2.5	22.4/4.5	48.4/8.3	100/20.6
P-value	**0.010**	**0.006**	**0.018**	**0.021**	0.44	0.22
Hypertension						
No	27.7/4.1	53.5/9.3	21.3/4.1	38.1/9.0	55.2/9.6	132.6/26.1
Yes	30.3/3.0	64.4/7.4	23.5/3.1	51.9/8.3	46.3/7.8	104.6/21.1
P-value	0.82	0.65	0.99	0.80	0.43	0.41
Presence of metastasis						
No	28.1/2.4	56.8/5.4	18.7/2.3	37.4/5.4	52.8/5.8	118.9/15.4
Yes	42.7/4.9	95.5/10.0	45.4/5.5	101.3/15.8	46.1/14.9	120.6/44.5
P-value	**<.001**	**<.001**	**<.001**	**<.001**	0.72	0.86

Similarly, in the ccRCC subset, significant associations were detected between primary tumor TfR1 levels and a metastatic stage, lower BMI, lower hemoglobin and history of anemia and iron supplementation; and with greater differences than detected among all RCC patients (Table 2). Although a trend towards association between smoking, an adverse prognostic risk factor, and ccRCC tumor TfR1 H-score did not reach significance (p = 0.053), smoking was significantly associated with a higher ccRCC tumor TfR1 maximum staining intensity (MSI) (mean 1.3 for smoking history *vs*. mean 1.0 for no smoking history; p = 0.022). Highest ccRCC tumor levels of TfR1 were detected in patients with iron-deficient anemia, who had on average 5-fold higher levels than patients without iron-deficient anemia (Table 2).

Similar to ccRCC primary tumors, TfR1 levels in non-ccRCC primary tumors were significantly higher in anemic patients, with highest levels in iron-deficient anemic patients (Table 2). In contrast, neither metastatic stage nor other adverse clinical features were associated with non-ccRCC primary tumor TfR1 levels (Table 2).

### Association of renal cell tumor TfR1 levels with tumor pathology

**Figure 3:**
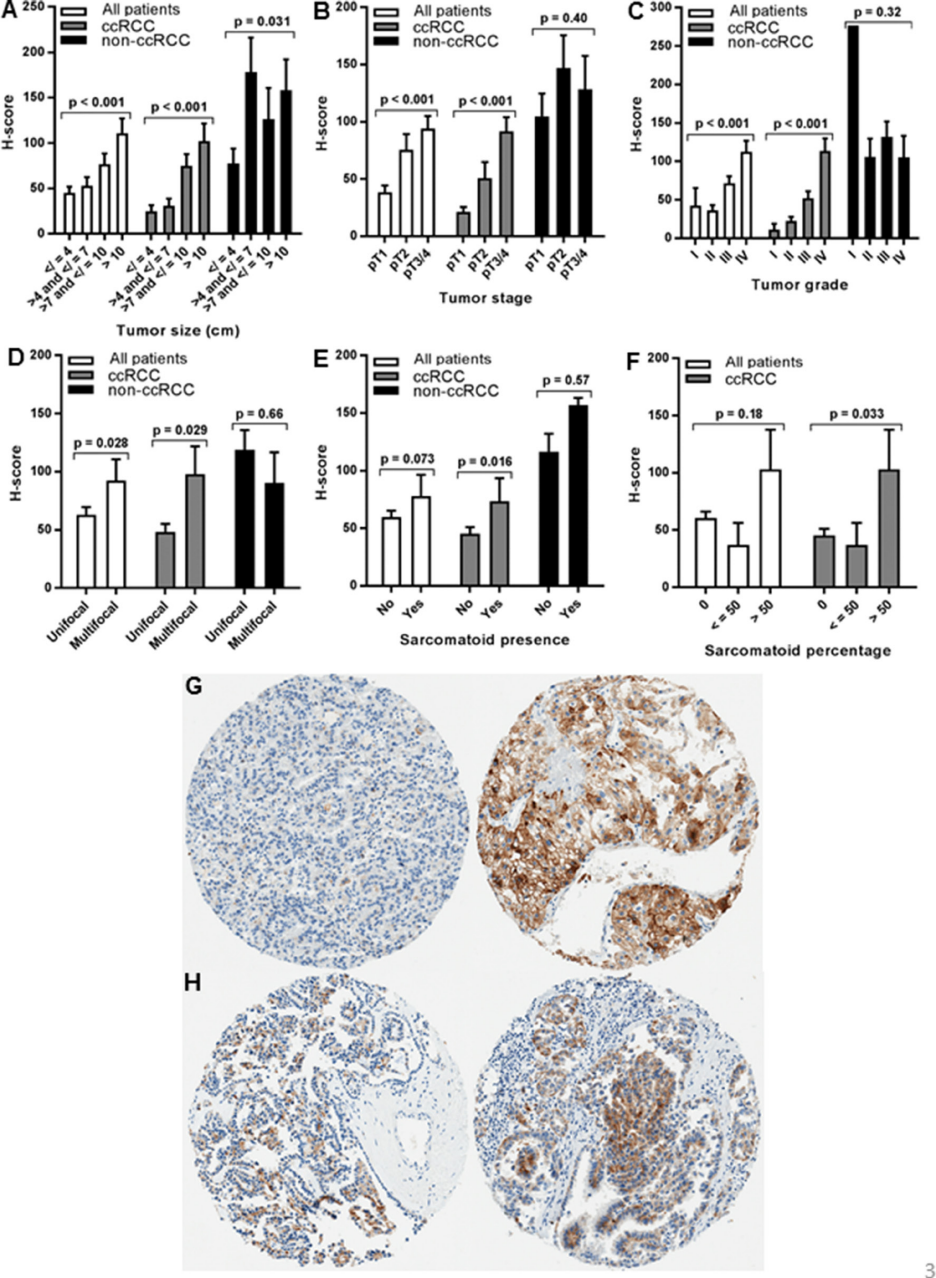
Association of primary tumor TfR1 H-score with renal cell tumor pathology Primary tumor TfR1 level (H-score) was measured by IHC using renal cell tumor patient TMAs and tested for association with pathologic features of primary tumors, including **(A)** tumor size (largest diameter), **(B)** tumor stage, **(C)** tumor grade, **(D)** tumor number (focality), **(E)** presence of sarcomatoid dedifferentiation, and **(F)** tumor percentage of sarcomatoid dedifferentiation. Only 1 grade I primary tumor was available for the non-ccRCC subset (C), and tumor percentages for two non-ccRCC patients with sarcomatoid dedifferentiation were unknown (F). Representative tissue core images of low grade/stage (left) and high grade/stage (right) primary tumors are shown from **(G)** ccRCC and **(H)** pRCCpatients.

RCC primary tumor TfR1 levels were tested for association with adverse pathologic features (Figure 3, Supplementary Figure 1). TfR1 upregulation in primary tumors was strongly associated with tumor size, stage and grade (p<0.001 each). Mean TfR1 H-scores in large (>10 cm) or grade IV tumors were similar to those of metastatic lesions (Figure 3A, 3C). TfR1 expression was also significantly increased in multifocal tumors (Figure 3D). A trend towards association between primary tumor TfR1 H-score and sarcomatoid dedifferentiation was non-significant (p = 0.073, Figure 3E), however TfR1 staining intensity was significantly associated with sarcomatoid dedifferentiation (mean MSI= 1.9 for present vs. 1.2 for absent, p = 0.004) and the tumor percentage of sarcomatoid involvement (mean MSI = 1.2 for 0%; 1.3 for 1-50%, 2.3 for >50%; p = 0.013). Similar findings were observed whether or not benign oncocytoma patients were included in analyses (data not shown).

Among the ccRCC primary tumor subset, similar significant associations between primary tumor TfR1 levels and all adverse pathologic features (tumor size, stage, grade, multifocality, sarcomatoid presence/percentage) were observed; and were of greater magnitude than observed in the full patient cohort (Figure 3, Supplementary Figure 1). Each stepwise increase in ccRCC tumor stage or grade was associated with an approximate doubling of the tumor TfR1 H-score. Grade IV tumors had >10-fold higher TfR1 levels than grade I tumors; pT3/pT4 tumors had >4-fold higher levels than pT1 tumors; large tumors (>10 cm) had >4-fold higher levels than small (<4 cm) tumors.

In contrast to ccRCC tumors, no significant associations between non-ccRCC primary tumor TfR1 levels and adverse pathologic features were detected, with the exception that larger non-ccRCC tumors (>4 cm) had approximately twice as high TfR1 levels as smaller (<4 cm) non-ccRCC tumors (Figure 3A).

### Association of primary tumor TfR1 levels with RCC patient survival

**Figure 4:**
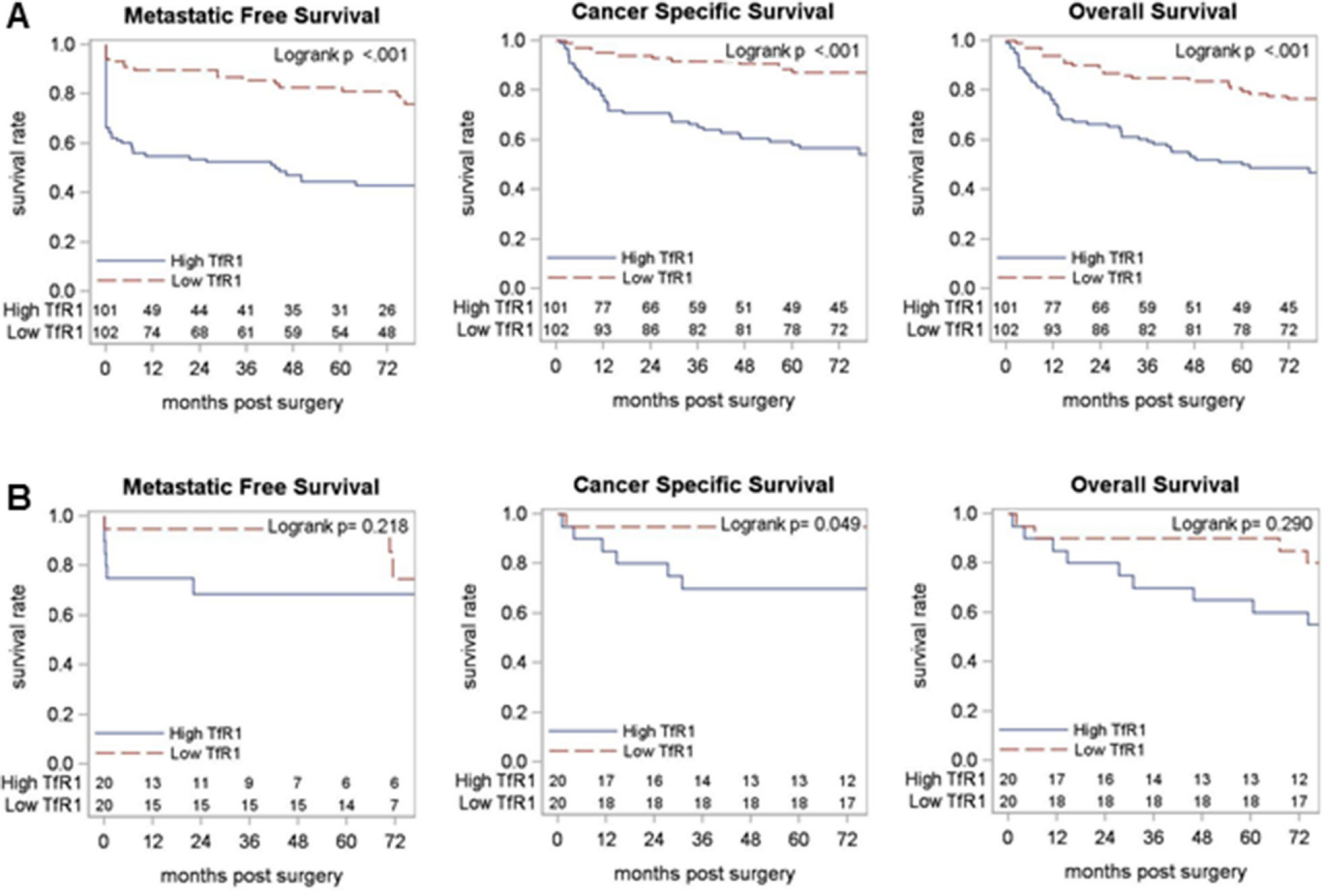
Association of primary tumor TfR1 levels with RCC patient survival TfR1 level (H-score) measured by IHC in RCC primary tumors was dichotomized at the median for **(A)** ccRCC and **(B)** non-ccRCC patient subsets and tested for association with (left to right) metastasis-free survival, cancer-specific survival and overall survival, using Kaplan–Meier methodology.

We next looked at the relationship between primary tumor TfR1 levels and patient survival outcomes (Figure 4, Table 3). In ccRCC patients, primary tumor TfR1 levels (either H-score or PTP) were strongly associated with a shorter time to metastasis (p<0.001), cancer-specific death (p<0.001) or death from any cause (p<0.001) (Figure 4A). In non-ccRCC patients, primary tumor TfR1 levels were associated with significantly worse cancer-specific survival, but not metastasis-free survival or overall survival (Figure 4B). In multivariable analyses adjusting for tumor pathology (and age for overall survival analyses), ccRCC primary tumor TfR1 level remained significantly associated with metastasis-free and overall survival outcomes (p<0.05 each); with a strong trend towards a significant independent association with cancer-specific survival (p=0.055). Similar multivariable analyses were not performed among non-ccRCC patients due to an inadequate number of metastasis/death events (Table 3).

**Table 3:** Association between primary tumor TfR1 level and RCC patient survival outcomes

TfR1 protein level (PTP or H-score)		Metastasis				Cancer-Specific Mortality				All-Cause Mortality			
		Univariate		Multivariable		Univariate		Multivariable		Univariate		Multivariable	
		HR (95% CI)	P-value	HR (95% CI)	P-value	HR (95% CI)	P-value	HR (95% CI)	P-value	HR (95% CI)	P-value	HR (95% CI)	P-value
ccRCC	PTP	3.44 (2.11, 5.59)	**<.001**	1.53 (0.87, 2.69)	0.14	4.51 (2.48, 8.20)	**<.001**	1.79 (0.93, 3.43)	0.080	2.71 (1.77, 4.17)	**<.001**	1.52 (0.95, 2.43)	0.080
	H-score	3.63 (2.19, 6.00)	**<.001**	1.82 (1.03, 3.21)	**0.041**	4.47 (2.42, 8.24)	**<.001**	1.90 (0.99, 3.67)	0.055	2.69 (1.74, 4.15)	**<.001**	1.61 (1.00, 2.59)	**0.048**
Non-ccRCC	PTP	1.94 (0.49, 7.65)	0.28	NP	NP	2.61 (0.53, 12.90)	0.17	NP	NP	0.95 (0.34, 2.65)	0.91	NP	NP
	H-score	2.16 (0.55, 8.52)	0.22	NP	NP	4.60 (0.69, 30.73)	**0.049**	NP	NP	1.69 (0.60, 4.76)	0.29	NP	NP

Hazard ratios (HR) refer to TfR1 levels above the median. NP = not performed.

### Association of metastasis TfR1 levels with RCC patient clinicopathologic features and survival

Similar analyses were performed to test the association between metastasis TfR1 levels and patient clinical features, primary tumor pathology and survival outcomes. There was no significant association between TfR1 levels in metastases and clinical or pathologic features, with the exception of primary tumor grade (Supplementary Figure 2). Metastasis TfR1 levels were not associated with cancer-specific or overall survival outcomes (Supplementary Table 3).

### Association of benign kidney TfR1 levels with renal cell tumor patient clinical features and pathology

**Figure 5:**
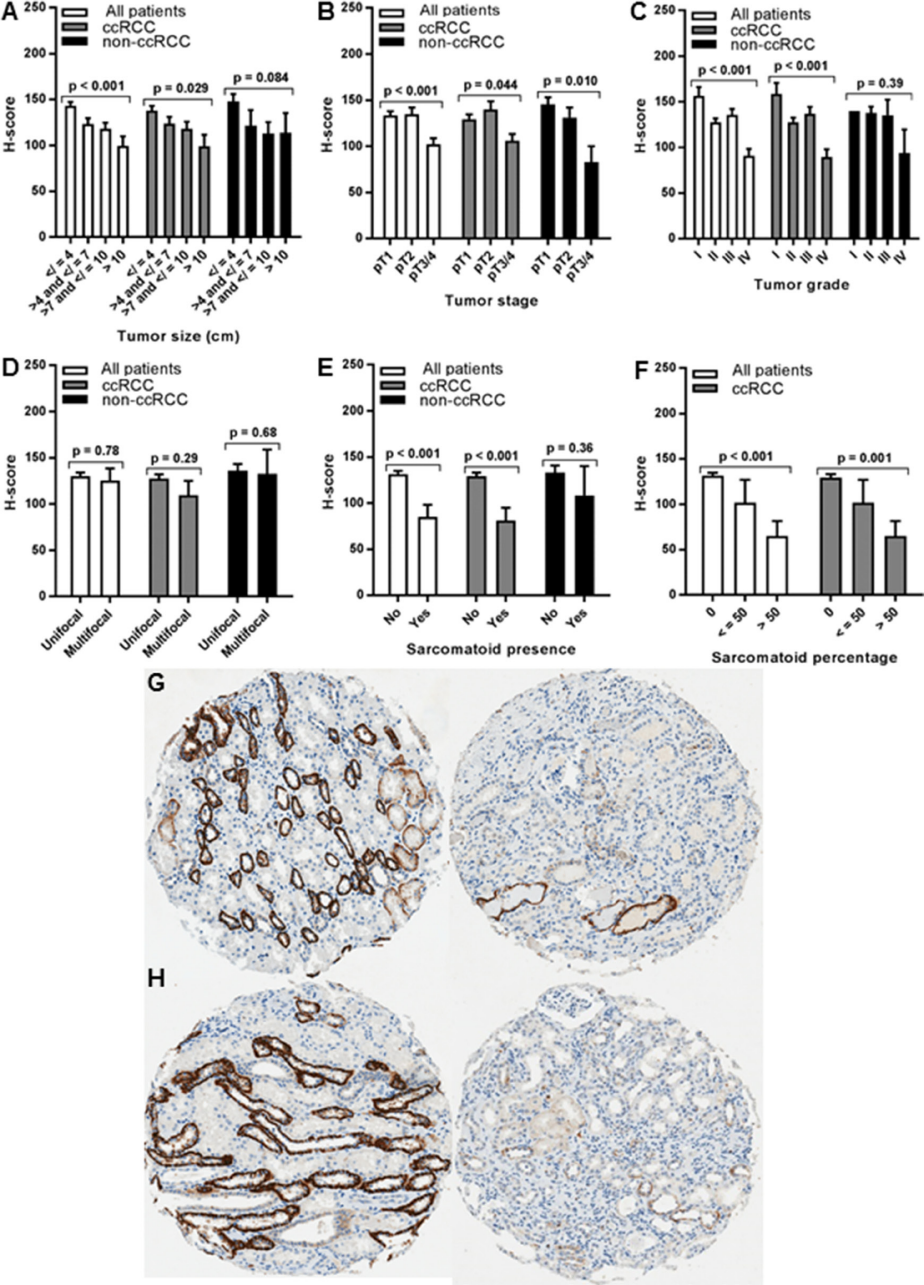
Association of normal kidney TfR1 H-score with RCC tumor pathology Normal (non-neoplastic) kidney TfR1 level (H-score) was measured by IHC using renal cell tumor patient TMAs and tested for association with pathologic features of renal cell primary tumors, including **(A)** tumor size (largest diameter), **(B)** tumor stage, **(C)** tumor grade, **(D)** tumor number (focality), **(E)** presence of sarcomatoid dedifferentiation, and **(F)** tumor percentage of sarcomatoid dedifferentiation. Only 1 primary tumor in the non-ccRCC subset was grade I (C), and tumor percentages for two non-ccRCC patients with sarcomatoid dedifferentiation were unknown (F). Representative tissue core images of normal kidney tissues are shown from **(G)** ccRCC and **(H)** pRCC patients with low grade/stage (left) or high grade/stage (right) primary tumors.

In addition to TfR1 levels in primary tumors and metastases, we tested TfR1 levels in benign kidney for association with renal cell tumor patient clinical features and pathology. Anemia and an extensive smoking history were each associated with significant decreases in benign kidney TfR1 levels (Table 4), in contrast to significant increases in primary tumor TfR1 levels (Table 2). In addition, TfR1 decreases in benign kidney correlated with a history of hypertension. Also opposite of observations in primary tumors, TfR1 expression in benign kidney was significantly reduced with RCC tumor progression (Figure 5, Supplementary Figure 3), based on size (p<0.001), grade (p<0.001), stage (p<0.001), sarcomatoid presence (p<0.001) and sarcomatoid percentage (p<0.001). Similar findings were observed whether or not benign oncocytoma patients were included in analyses (data not shown). Similar associations were also observed for the ccRCC subtype and to a lesser degree (tumor stage only) for non-ccRCC subtypes (Figure 5).

**Table 4:** Association between benign renal epithelial TfR1 level and renal cell tumor patient clinical features

	All Patients		ccRCC Patients		Non-ccRCC patients	
	TfR1 PTP	TfR1 H-score	TfR1 PTP	TfR1 H-score	TfR1 PTP	TfR1 H-score
	(mean/SE)	(mean/SE)	(mean/SE)	(mean/SE)	(mean/SE)	(mean/SE)
Age, years						
<= 60	46.6/1.7	124.8/4.8	47.7/2.0	127.5/5.7	47.0/3.0	128.7/9.9
> 60	46.8/1.6	125.8/4.5	44.6/1.9	119.6/5.2	51.4/4.	134.8/10.2
P-value	0.95	0.89	0.15	0.14	0.20	0.37
Gender						
Male	46.0/1.4	124.2/3.9	45.0/1.6	121.2/4.7	50.0/2.6	134.2/6.7
Female	48.0/2.1	127.1/6.0	48.4/2.5	128.1/6.8	47.8/5.3	126.8/16.2
P-value	0.42	0.55	0.27	0.32	0.93	0.77
Race						
White	46.5/1.2	124.7/3.4	46.0/1.4	123.1/4.0	48.6/2.5	131.1/6.7
Black	49.8/4.2	133.4/13.1	52.3/5.5	145.7/17.2	51.2/8.5	133.7/26.6
P-value	0.65	0.80	0.45	0.30	0.47	0.73
Body Mass Index, kg/m^2^						
< 30	45.8/1.7	124.7/4.9	44.3/1.9	120.9/5.6	50.2/3.5	132.6/10.0
>= 30	48.0/1.8	128.1/5.1	47.9/2.1	127.0/5.7	45.1/3.8	123.1/11.2
P-value	0.38	0.48	0.19	0.29	0.25	0.32
Smoking History						
Never	47.3/1.8	127.8/5.3	45.9/2.3	123.0/6.4	49.9/3.4	138.8/10.0
Any	45.9/1.6	122.4/4.4	46.2/1.8	123.4/5.0	48.3/3.6	124.3/9.8
P-value	0.19	0.17	0.64	0.64	0.68	0.29
Pack Years						
0	47.3/1.8	127.8/5.3	45.9/2.3	123.0/6.4	49.9/3.4	138.8/10.0
<= 30	48.8/2.5	129.0/6.9	48.4/3.0	128.8/8.4	53.3/4.0	137.0/11.5
> 30	39.5/2.8	106.9/7.9	39.4/2.9	106.6/8.2	29.3/5.2	75.5/16.5
P-value	**0.011**	**0.023**	0.068	0.11	**0.018**	**0.027**
Iron Supplement Medication						
No	46.6/1.3	125.3/3.7	45.5/1.5	121.6/4.3	48.3/2.5	129.9/7.2
Yes	44.2/4.0	113.6/11.3	45.2/3.3	115.2/7.8	41.7/14.0	109.4/42.1
P-value	0.52	0.28	0.81	0.47	0.58	0.58
Anemia						
No	51.8/1.8	140.0/4.9	50.8/2.3	135.5/6.1	52.9/3.1	147.0/8.9
Yes	41.7/2.5	110.3/7.3	38.6/3.0	102.0/8.7	48.2/3.3	123.9/7.6
P-value	**0.004**	**<.001**	**0.006**	**0.003**	0.18	0.057
Iron-Deficient Anemia						
No	50.0/1.7	135.1/4.7	48.5/2.2	129.5/5.9	50.9/2.4	139.1/7.4
Yes	44.6/5.7	114.7/18.6	44.1/7.1	112.4/22.8	46.7/0.0	125.0/15.0
P-value	0.28	0.14	0.46	0.28	0.46	0.37
Hemoglobin, g/dL						
< 13.3						
	44.2/2.2	117.6/6.6	41.8/5.2	110.3/15.1	51.0/3.3	131.8/9.2
>= 13.3	51.0/2.1	138.0/5.5	47.0/2.1	125.1/5.6	50.8/3.2	142.7/8.9
P-value	**0.042**	**0.011**	0.44	0.26	0.80	0.21
Hypertension						
No						
	49.9/2.3	135.9/6.6	40.6/2.5	107.7/7.5	54.7/4.2	49.3/12.6
Yes	44.6/1.6	118.7/4.3	50.8/2.7	135.2/7.0	43.9/3.4	117.1/9.2
P-value	**0.041**	**0.015**	**0.021**	**0.009**	0.084	**0.044**
Presence of metastasis						
No						
	48.2/1.2	130.7/3.5	48.5/2.7	131.3/7.5	50.3/2.5	135.6/7.0
Yes	39.4/3.0	98.6/8.2	43.7/1.8	115.3/5.0	36.1/9.4	90.6/25.9
P-value	**0.013**	**<.001**	0.095	**0.04**	0.15	0.094

### Association of benign kidney TfR1 levels with RCC patient survival

**Figure 6:**
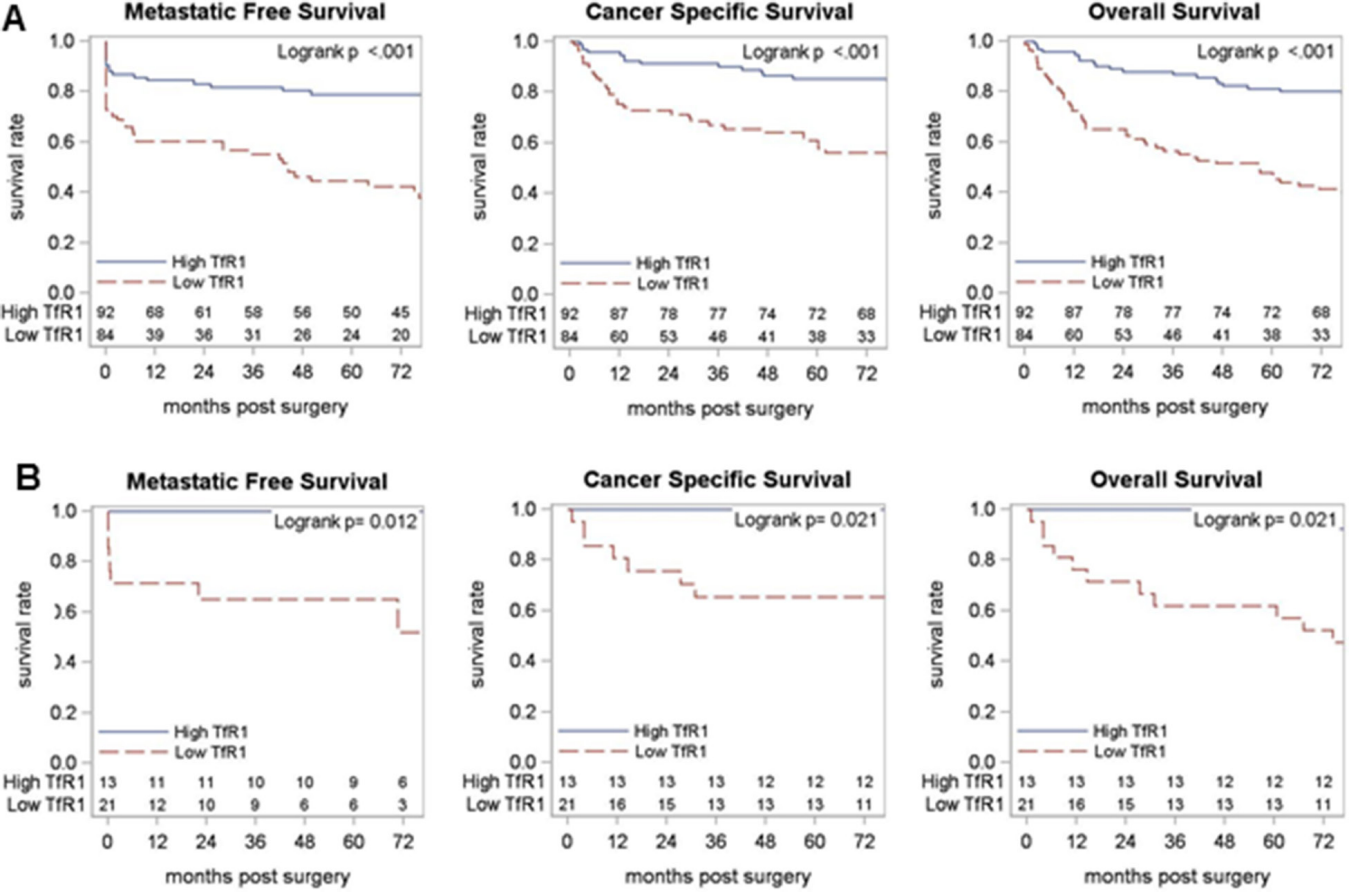
Association of normal kidney TfR1 levels with RCC patient survival TfR1 level (H-score) measured by IHC in normal (non-neoplastic) kidney tissues from RCC patients was dichotomized at the median for **(A)** ccRCC and **(B)** non-ccRCC patient subsets and tested for association with (left to right) metastasic-free survival, cancer-specific survival and overall survival, using Kaplan–Meier methodology.

In survival analyses, lower TfR1 expression in benign kidney tissue of ccRCC patients was associated with a significantly shorter time to metastasis (p<0.001), cancer-specific death (p<0.001) and death due to any cause (p<0.001) (Figure 6A, Table 5). Significant associations with survival outcomes were similarly observed in non-ccRCC patients (Figure 6B, Table 5). In multivariable analyses adjusting for tumor pathology (and also for age in overall survival analyses), lower TfR1 levels in benign kidney remained independently associated with worse cancer-specific and overall survival among ccRCC patients (Table 5). Similar multivariable analyses were not performed among non-ccRCC patients due to an inadequate number of metastasis/death events.

**Table 5:** Association between benign renal epithelial TfR1 protein level and RCC patient survival outcomes

TfR1 protein level (PTP or H-score)		Metastasis				Cancer-Specific Mortality				All-Cause Mortality			
		Univariate		Multivariable		Univariate		Multivariable		Univariate		Multivariable	
		HR (95% CI)	P-value	HR (95% CI)	P-value	HR (95% CI)	P-value	HR (95% CI)	P-value	HR (95% CI)	P-value	HR (95% CI)	P-value
ccRCC	PTP	0.48 (0.29, 0.80)	**0.002**	0.78 (0.44, 1.37)	0.39	0.39 (0.22, 0.70)	**<.001**	0.58 (0.31, 1.10)	0.090	0.43 (0.27, 0.68)	**<.001**	0.64 (0.39, 1.05)	0.080
	H-score	0.36 (0.22, 0.61)	**<.001**	0.64 (0.36, 1.16)	0.14	0.30 (0.17, 0.54)	**<.001**	0.50 (0.26, 0.97)	**0.039**	0.32 (0.20, 0.51)	**<.001**	0.50 (0.30, 0.84)	**0.009**
Non-ccRCC	PTP	0.20 (0.03, 1.29)	**0.033**	NP	NP	0.24 (0.04, 1.64)	0.14	NP	NP	0.20 (0.05, 0.83)	**0.008**	NP	NP
	H-score	0.07 (0.00, 1.56)	**0.012**	NP	NP	0.09 (0.00, 1.89)	**0.021**	NP	NP	0.24 (0.06, 0.99)	**0.021**	NP	NP

Hazard ratios (HR) refer to TfR1 levels above the median. NP = not performed.

### TfR1 expression in ccRCC and benign renal epithelial cell lines

**Figure 7:**
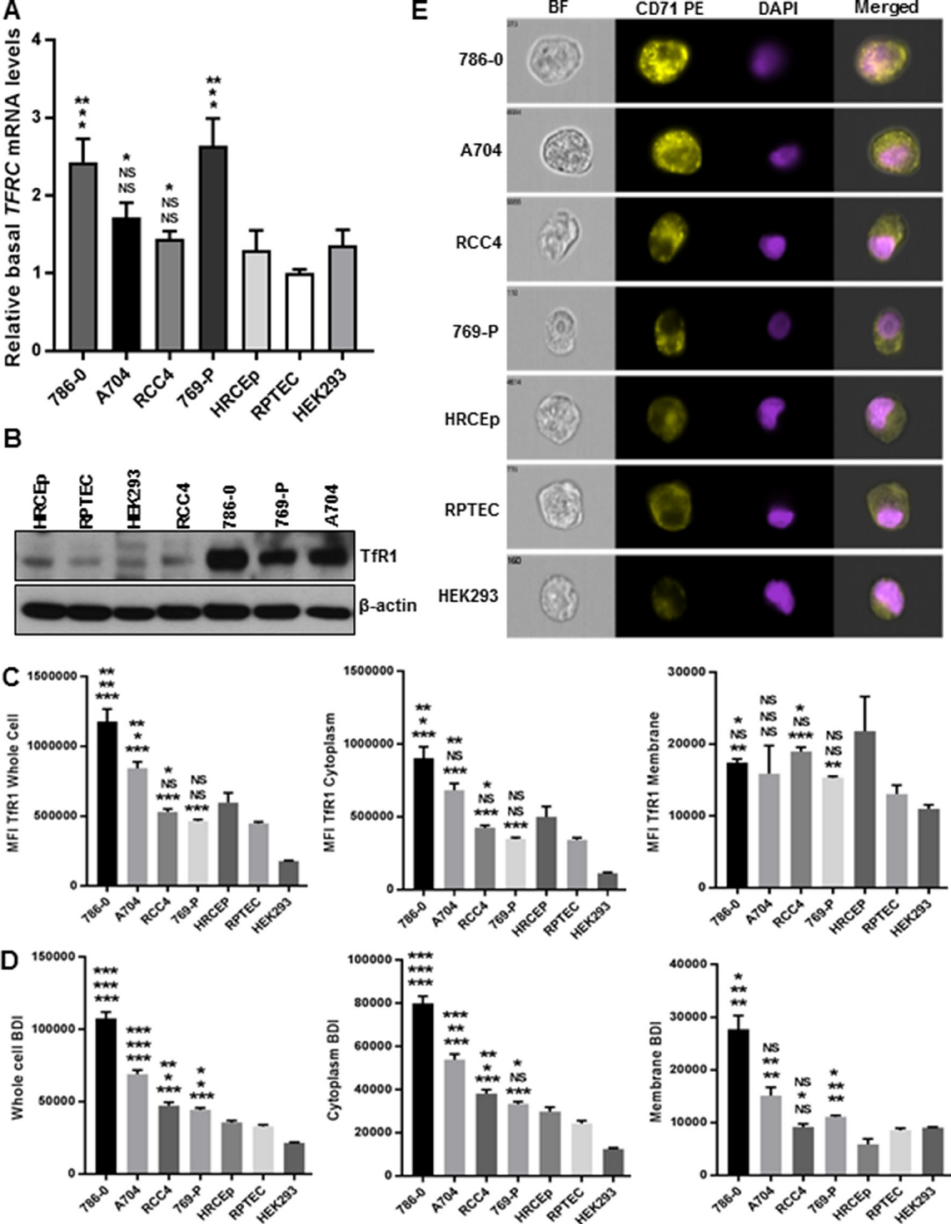
TfR1 expression in ccRCC and benign renal epithelial cell lines TfR1 expression was compared between benign renal cell lines (RPTEC, HRCEp, HEK293) and ccRCC cell lines (RCC4, 786-0, 769-P, A704) using different approaches. **(A)** Expression of *TFRC* mRNA transcript measured using quantitative RT-PCR and normalized to RPTEC. **(B)** Expression of total TfR1 protein depicted in a representative Western blot. **(C)** Mean fluorescence intensity (MFI) of TfR1 staining in whole cell vs. cytoplasmic vs. membranous fractions using imaging flow cytometry with morphology-based masking. **(D**) Bright detail intensity (BDI) of TfR1 staining using imaging flow cytometry with morphology-based masking to specifically measure punctate staining foci/clusters in whole cell vs. cytoplasmic vs. membranous fractions. **(E)** Representative images of TfR1 staining in ccRCC and benign renal cell lines obtained using imaging flow cytometry. Statistical analysis was performed using a Student’s *t*-test. Data are presented as means ± SEM from at least 3 independent experiments; ^*^, p<0.05; ^**^, p<0.01; ^***^, p<0.001; NS = non-significant for comparison to the RPTEC cell line (top asterisk), HRCEp (middle asterisk) and HEK293 (bottom asterisk).

We next sought to evaluate TfR1 expression in a panel of benign renal and ccRCC cell lines cultured under iron-replete conditions. Analysis of *TFRC* gene expression indicated generally higher transcript levels in ccRCC cell lines than benign renal cell lines (Figure 7A). Western blot demonstrated higher TfR1 protein expression in three of the four ccRCC cell lines compared to benign renal cell lines (Figure 7B). Imaging flow cytometry performed to quantitatively evaluate TfR1 localization confirmed a mixture of cytoplasmic and membranous expression. Furthermore, ccRCC cell lines tended to express higher levels of TfR1 in the whole cell and in the cytoplasm compared to benign renal cell lines (Figure 7C, 7E). Abundant punctate foci/clusters of TfR1 staining were visualized in both the cytoplasmic and membranous compartments of all cell lines, and were of significantly higher staining intensity in ccRCC cell lines compared to benign cell lines (Figure 7D, 7E).

## DISCUSSION

Iron has long been implicated in carcinogenesis, although its importance and precise role remain poorly understood. Alterations in iron uptake may play a unique role in RCC, since this cancer is often defined by a dysregulated VHL/HIF-α pathway that governs intracellular iron levels. Consistent with this hypothesis, occupations in the iron/steel industry and medical conditions at risk for systemic iron overload are each reported to have elevated rates of RCC diagnosis [31, 32, 52-54]. Furthermore, chronic systemic iron administration to rodents causes renal tumorigenesis that mimics key features of human RCC. Yet despite these compelling observations, the role of iron uptake in human RCC has received scarce investigation to date.

TfR1 is the primary receptor mediating intracellular iron uptake, and characterization of its expression during RCC tumorigenesis and progression is therefore fundamental to understanding the role of iron metabolism in this cancer. Interestingly, polymorphisms have been identified within miRNA binding sites of the human TfR1 gene, *TFRC*, that increase the risk of RCC [55]. However, TfR1 expression levels in RCC patients have remained unclear. Most RCC tumors, particularly ccRCC, are characterized by accumulation of HIF-α transcription factors that are activators of TfR1 expression [45-47]. HIF-2α protein can be increased by iron uptake due to an IRE motif within the HIF-2α transcript [51], and higher HIF-2α protein levels correlate with ccRCC progression [50]. These observations provide a mechanistic basis for increases in TfR1 protein during RCC progression, particularly for the ccRCC subtype.

The current study investigates TfR1 expression and its prognostic significance in renal cell tumor patients. Several prior studies in various cancer types have concluded no association between TfR1 expression and cancer progression [56-60]. In contrast, the current study found TfR1 protein levels in RCC primary tumors, and particularly the ccRCC subtype, to be strongly associated with adverse RCC pathology, including primary tumor size, stage, grade, number and sarcomatoid dedifferentiation. Significantly higher tumor TfR1 levels were also detected in patients with a lower BMI, smoking history and anemia (particularly iron-deficient anemia and patients on iron supplementation), all clinical features associated with worse RCC patient outcomes [61-64]. Survival analyses confirmed an association between higher ccRCC primary tumor TfR1 levels and shorter times to patient metastasis and mortality, independent of tumor pathology. These findings indicate that higher primary tumor TfR1 levels correlate with more aggressive clinicopathologic features and a worse overall prognosis, particularly for the ccRCC subtype. Relatively high TfR1 levels detected in this study among several ccRCC cell lines may reflect their origins from advanced primary tumors [65].

While studies of other cancer types have identified higher TfR1 levels in malignant versus benign tissues [66-68], increased expression has typically not correlated with cancer progression [56-60, 68-71]. Hence, TfR1 protein may have a role in ccRCC tumor progression that is largely unique to this cancer type. Although a similar conclusion cannot be reached for non-ccRCC subtypes based on our current data, our finding that higher non-ccRCC primary tumor levels of TfR1 correlated with cancer-specific mortality suggests a similar role as in ccRCC. Larger studies are warranted to more definitively investigate the prognostic significance of TfR1 expression in individual non-ccRCC subtypes.

The mechanism by which higher primary tumor TfR1 levels might contribute to RCC progression is unclear. Our detection of abundant TfR1 protein distributed in clustered foci among ccRCC cell lines might indicate increased trafficking to and/or from the membrane, however additional study is needed. Increased iron uptake as a result of higher TfR1 activity might promote tumor progression by increasing HIF-2α protein through IRP1 inactivation [51]. This mechanism would likely require a defective VHL/PHD axis, as is present in ccRCC, since iron uptake otherwise reduces HIF-2α levels by activating iron-dependent PHD enzymes [3]. Future studies that assess the correlation of HIF-α expression levels with TfR1 expression levels in RCC tumors will be helpful to support or challenge this mechanistic model.

Alternatively, TfR1 upregulation and increases in intracellular iron might have a selective advantage for RCC progression due to HIF-α-independent mechanisms. The requirement of iron to catalyze DNA repair [21] might be critical for tumor progression, since ccRCC tumors are known to harbor recurrent mutations in several chromatin remodeling/DNA repair genes [4], and the requirement of efficient DNA repair in the setting of such mutations is well described [72]. In addition, the established role of iron in oxidative stress induction might also contribute to progression of RCC patient tumors, which harbor recurrent mutations in known redox genes [6, 14-17]. Oxidative stress is known to mediate the carcinogenic effect of iron in the rodent kidneys [73], however its role in tumor progression is unclear. The hypothesis that iron uptake promotes RCC progression through its classic catalytic role in DNA synthesis and cell division is challenged by our finding of highest TfR1 protein levels in chRCC, the most indolent RCC subtype; in addition to relatively high TfR1 levels in benign renal oncocytoma.

Anemia is a common characteristic in advanced RCC patients and typically manifests with low serum iron levels. An alternative explanation for current findings is that higher primary tumor TfR1 protein levels are a compensatory response to lower serum iron levels and IRP1-mediated TfR1 transcript stabilization [39]. Consistent with this explanation, we observed an association between patient anemia and higher tumor TfR1 levels in both ccRCC and non-ccRCC patients. Similarly, Tong et al. has attributed high TfR1 expression in a pRCC cell line to an intracellular iron-deficient state [74]. However, we also observed a decrease (rather than increase) in TfR1 levels in benign kidney tissue of anemic patients, indicating that lower serum iron levels alone cannot account for higher tumor TfR1 levels.

TfR1 may provide a useful therapeutic target for advanced RCC patients. Currently approved clinical therapeutics that target indirectly the VHL/HIF-α axis, which include angiogenesis and mTOR kinase inhibitors, achieve only modest improvements in RCC patient survival [13]. Direct targeting of HIF-2α is under investigation with promising early results [75], and certain drugs that lower HIF-2α levels have been suggested to work through reduction of iron levels [47]. Our laboratory and others have shown that iron deprivation can effectively suppress HIF-2α expression in ccRCC cells *in vitro*, however no clinical investigation has explored iron uptake targeting in RCC patients to our knowledge [51, 76]. Encouraging outcomes have been reported in neuroblastoma patients treated with intravenous iron chelation as a chemotherapy adjuvant, and in advanced hepatocellular carcinoma patients as sole therapy [77, 78]. Orally administered iron chelator drugs are now available, but pilot studies in cancer patients have been limited by low grade toxicity [79]. Development of novel approaches to lower iron levels selectively in cancer cells, including by targeting tumor TfR1, holds promise and warrants future study [80].

Two additional findings of interest in the current study are 1) highest TfR1 levels in the non-gravid human body are found in the benign kidney, and 2) decreases in benign kidney TfR1 levels correlate with RCC tumor progression. Regarding the first finding, only placenta, which transports large amounts of Tf to the developing fetus [81], had higher TfR1 levels than kidney among 36 normal body tissues evaluated. Renal TfR1 expression localized specifically to the tubule epithelium, the presumed site of RCC tumorigenesis. High benign renal TfR1 levels were unexpected since prior studies in other cancer types report minimal TfR1 expression in matched benign tissues [59, 66, 68, 82]. These results suggest a unique role for iron metabolism in the kidney, as supported by other observations such as the renal specificity of erythropoietin hormone production [25]; the critical role of Tf for induction of renal tubular and glomerular formation during embryogenesis [83]; and the highest body tissue levels of IRP1 in the rodent kidney [84]. The functional significance of iron uptake into renal tubule epithelium is unclear, but is probably unrelated to cell division because renal epithelium is non-proliferative in the absence of acute renal injury [85]. The polarity of renal tubule TfR1 expression along the basal membrane surface suggests iron is drawn into tubules from adjacent stromal tissue, perhaps for excretion at the luminal epithelial surface into urine. Such a role would be unnecessary to maintain in non-polarized RCC cells, perhaps explaining lower TfR1 levels in tumors. Given the diversity of potential cellular functions of iron, we suspect that the primary role of iron uptake in benign kidney is distinct from its role in RCC tumors; and that high baseline TfR1 levels in kidneys might simply make the renal epithelium prone to iron overload in situations of high systemic exposure, accounting for the renal specificity of rodent or human cancers in situations/conditions of chronic iron overload [31, 32, 52-54]. Consistent with this hypothesis, systemic administration of iron to rodents leads to detectable iron deposits in only a few organs, namely the kidney, heart and liver [86], each of which demonstrated relatively high TfR1 levels in the present study.

To the best of our knowledge, this report is the first to identify independent prognostic significance of a benign renal tissue protein biomarker for RCC patients. Opposite to increases in primary tumor TfR1 levels during progression, benign renal TfR1 levels decreased in association with adverse clinical prognostic characteristics, RCC tumor progression and patient mortality. The opposite TfR1 alterations in benign kidney and primary tumors during tumor progression might reflect contrasting tissue responses to iron accumulation due to VHL/PHD status. Whereas iron accumulation in VHL-deficient ccRCC tumor cells might increase HIF-2α through IRP1 deactivation and in turn promote *TFRC* transcription, iron accumulation in VHL wild-type tissues would be expected to decrease HIF-2α through PHD activation and lead to *TFRC* downregulation. Whether iron accumulation occurs with RCC tumorigenesis is unknown but it is a well described early event in other renal pathologies [85]. Studies are ongoing in our laboratory to address this question.

Metastatic relapse after nephrectomy for localized disease is estimated to occur in at least 20% of RCC patients [11]. Currently, nephrectomy patient prognosis is estimated based on tumor histopathology, and validated molecular markers that increase the accuracy of prognostication are lacking. Although small, non-invasive RCC tumor patients who undergo partial or radical nephrectomy (tumor stage pT1a) have an outstanding prognosis, many of these patients can be managed conservatively with active surveillance rather than surgery, and most patients undergoing nephrectomy in the contemporary era have more advanced cancers (>pT1b) for which postoperative recurrence and mortality are frequent [11]. Our results suggest that TfR1 protein in either the primary tumor or benign kidney may provide a novel biomarker for nephrectomy patients with prognostic value independent of primary tumor pathology, particularly for ccRCC patients. Prospective studies are need to determine whether measurement of tumor or kidney levels of TfR1 in biopsied or surgically resected specimens might assist physicians in postoperative management of nephrectomy patients, including high-risk patient identification for adjuvant therapy trials. Although TfR1 levels in metastasis tissues did not correlate with survival duration in this study, response to individual therapeutic regimens for metastatic disease was not addressed and warrants future investigation.

## CONCLUSION

Although iron has long been implicated in carcinogenesis, its role remains poorly understood. We suspect that iron uptake has a unique role in RCC, given the presence of common alterations in the VHL/HIF-α pathway that regulates iron levels; in addition to compelling observations from rodent experiments and epidemiologic studies. The current investigation reveals that the primary iron uptake protein, TfR1, has highest body tissue levels in benign kidney, which underscores an important but still uncharacterized role for this protein in renal pathophysiology. Furthermore, alterations in TfR1 expression in the primary tumor and benign kidney correlate with RCC disease progression and patient mortality. These findings support that iron metabolism may play a critical role in RCC tumorigenesis and progression. In the future it will be critical to elucidate mechanisms of TfR1 regulation and iron metabolism in RCC patients, which may yield novel preventative strategies, biomarkers and targeted therapies for this disease.

## MATERIALS AND METHODS

### Patients and tissues

Institutional review board approval at Roswell Park Cancer Institute (RPCI) was obtained for this study. Informed consent was provided by all patients. A total of 574 paraffin-embedded formalin-fixed tissue specimens (primary renal cell tumor, matched benign kidney and/or metastasis) were studied from 287 patients who underwent radical or partial nephrectomy (N=268) and/or metastatectomy (N=74) for either RCC or benign renal oncocytoma between 1995 and 2008 at RPCI, a National Comprehensive Cancer Network institute. Deidentified clinicopathologic and survival data were obtained from a prospectively maintained RPCI nephrectomy patient database and the RPCI cancer patient registry. Primary tumor (T) stage and grade were assigned per updated guidelines of the American Joint Commission on Cancer and the International Society of Pathologists, respectively. Histologic subtype was assigned per recommendations of the World Health Organization criteria and analyzed as either ccRCC or non-ccRCC, the latter of which included pRCC, chRCC, unclassified RCC or rare RCC subtypes. Clinical data delivery and Honest Broker deidentification services were provided by the RPCI Clinical Data Network.

### Tissue microarray construction and immunohistochemistry (IHC)

Three TMAs were constructed from RPCI renal cell tumor patient tissues. Triplicate needle cores of 1.0 mm diameter were procured from representative areas of each tissue specimen. Each triplicate core was embedded in a separate paraffin block, generating a total of nine paraffin blocks for the three TMAs. Tissue blocks included cores from 14 different non-neoplastic body sites as internal staining controls. A slide section of 4 μm thickness was generated for IHC staining. Additionally, a 4 μm section from a separate TMA harboring 1.5 mm normal (benign) tissue cores from 102 different individuals was obtained from US Biomax, Inc (Derwood, MD, USA). A total of 36 different types of normal tissue (2-3 cores each) were represented on this TMA.

TfR1 immunostaining was performed using a Dako Omnis autostainer (Agilent Technologies, Santa Clara, CA, USA). TMA slides were deparaffinized with Clearify and rehydrated using graded alcohols. Target retrieval was performed using Flex TRS High pH (Agilent Technologies) for 30 minutes. Slides were incubated with mouse anti-human TfR1 antibody (Thermo-Fisher Scientific, Waltham, MA, USA) for 30 minutes at 1:50 dilution. Envision Flex Mouse Linker (Agilent Technologies) secondary antibody was applied for 10 minutes followed by Envision Flex/HRP labeled polymer (Agilent Technologies) for 30 minutes. Dab chromogen was applied for 5 minutes for visualization. Slides were counterstained with Hematoxylin for 8 minutes. TfR1 immunostaining was scored under a clinical genitourinary pathologist (BX) based on PTP score (0-100%) and staining intensity (0+ absent, 1+ low, 2+ moderate, 3+ high). Each core stain was summarized by a TfR1 H-score, equivalent to the product of the TfR1 PTP score and the TfR1 intensity score.

### Cell lines

All cell lines were obtained from the American Type Culture Collection (Rockville, MD, USA), with the exception of the RCC4 cell line which was obtained from the European Collection of Authenticated Cell Cultures General Cell Collection (Salisbury, UK). 786-0, 769-P, A704 and RCC4 cell lines were derived from ccRCC primary renal tumors and harbor known *VHL* mutations. The human renal cortical epithelium (HRCEp) cell line and renal proximal tubule epithelial cell (RPTEC) line are derived from benign human kidney. The HEK293 cell line is derived from benign human embryonic kidney. All cell lines were maintained *in vitro* in DMEM media supplemented with L-glutamine (4 mM), sodium pyruvate (110mg/L), glucose (4.5g/L) (Corning Cellgro, Manassas, VA, USA), penicillin-streptomycin (100U) (Corning Cellgro) and 10% fetal bovine serum (Seradigm, Radnor, PA, USA). Cultures were maintained at 37°C with 5% CO_2_. All cell lines were grown to 60-80% confluency prior to assay.

### RNA isolation and qPCR

Total RNA was isolated using a QIAshredder (Qiagen, Germantown, MD, USA) and RNase mini kit (Qiagen). Genomic DNA was digested using DNase I (Qiagen) as described in the on-column DNase digestion protocol from Qiagen. RNA concentration was determined using a Nanodrop 2000c (Thermo-Fisher Scientific). One microgram of RNA was used for reverse transcription using iScript cDNA synthesis kit (Bio-Rad, Hercules, CA, USA) according to the manufacturer’s instructions. Quantitative real-time PCR was performed using iTaq Universal SYBR Green Supermix as recommended by the manufacturer (Bio-Rad). The samples were analyzed on a CFX Connect Real-Time PCR Detection system (Bio-Rad) with the following parameters: 10 min at 95°C, 40 cycles at 95°C for 15 sec and 60°C for 1 min. Melting curves were obtained by increasing temperature from 55°C to 95°C by 0.5°C increments. ΔCt was calculated as the difference between the Ct value for TfR1 and the average of the Ct values for the B2M and DIMT1 housekeeping genes (ΔCt = CtTfR1 – Ct (B2M+DIMT1)/2), using a threshold cycle limit of 200 RFU. Fold change in expression between TfR1 and the housekeeping genes for each cell line (2^ΔCt^) was normalized to the RPTEC cell line. Primers were purchased from Integrated DNA Technologies (IDT, Coralville, IA). Primer sequences included *TFRC* forward: ACTTGCCCAGATGTTCTCAG; *TFRC* reverse: GTATCCCTCTAGCCATTCAGTG; *B2M* forward: GGCATTCCTGAAGCTGACAG; *B2M* reverse: TGGATGACGTGAGTAAACCTG; *DIMT1* forward: TGATGTAGTGCTGGAAGTTGG, *DIMT1* reverse: GTGCCCTGAACTCTTTTGTG.

### Western blot

Cell lines were lysed using RIPA buffer (Thermo-Fisher Scientific) supplemented with Halt^™^ protease inhibitor (Thermo-Fisher Scientific). Cell debris was removed after centrifugation at 4°C, and total protein concentration was determined using the DC protein assay (Bio-Rad). Electrophoretic separation of protein (12 μg/well) was performed using 4-15% gradient polyacrylamide gels (Bio-Rad). Separated protein was transferred for 18 hours at 4°C onto PVDF membranes (Bio-rad). Membranes were blocked for one hour at room temperature in TBS containing 0.1% tween (TBS-T) with 5% fat-free milk, followed by overnight incubation at 4°C with mouse anti-human TfR1 antibody (Thermo-Fisher Scientific) (1:500 dilution) or mouse anti-human β-actin antibody (Cell Signaling Technology, Danvers, MA, USA) (1:10,000 dilution) in 5% fat-free milk with TBS-T. Membranes were washed in TBS-T and incubated for 30 minutes at room temperature with a 1:2000 dilution of horseradish peroxidase-conjugated rabbit anti-mouse antibody (Cell Signaling Technology) in 5% milk with TBS-T. Protein signals were developed on X-ray film using the Pierce ECL Western blotting substrate (Thermo-Fisher Scientific) or the SuperSignal West Femto Maximum Sensitive Substrate (Thermo-Fisher Scientific). X-ray film of blots was digitized using an office scanner (Epson, Long Beach, CA, USA).

### Imaging flow cytometry

Cells were harvested using Accutase enzymatic solution (Sigma-Aldrich, St. Louis, MO, USA), washed and resuspended in 200 μL FACS buffer (PBS, 5% FBS, and 0.01% sodium azide), followed by incubation with 5 μL phycoerythrin-conjugated anti-human TfR1 antibody (BioLegend, San Diego, CA, USA) for 20 minutes at room temperature to stain for TfR1 membranous protein. Surface-stained cells were then washed and fixed for 10 minutes in 1 mL of 4% Formaldehyde solution (Polysciences, Inc. Warrington, PA, USA), followed by permeabilization for 20 minutes at room temperature using 200 μL of 0.01% Triton^™^ X-100 buffer (Sigma Aldrich, St Louis, MO, USA). After fixation/permeabilization, cells were re-incubated with phycoerythrin-conjugated anti-human TfR1 antibody for 20 minutes at room temperature to stain cytoplasmic TfR1 protein. Cells were then washed and resuspended into 30 μL FACS buffer, to which 10 μL DAPI (4’,6-Diamidino-2-Phenylindole)(Life Technologies, Carlsbad, CA, USA) at 5 μg/mL was added for 10 minutes for nuclear staining. Sample acquisition was performed using the ImageStreamX^™^ MKII imaging flow cytometer (Amnis, Seattle, WA, USA) with at least 10,000 events recorded per sample using INSPIRE software (Amnis). Sample analysis was conducted using IDEAS software v6.2. Morphology-based masks were created to distinguish between whole cell vs. cytoplasmic vs. membranous fractions of the cells. Mean fluorescence intensity (MFI) was then calculated using the morphology-based masks for the overall expression of TfR1 on these fractions of the cells. The Bright Detail Intensity (BDI) was measured to compute the intensity of localized or clustered TfR1 protein using an intensity threshold of ≤ 3 pixels in radius within the masked area for the whole cell, cytoplasm and membrane fractions.

### Statistics

Patient characteristics were summarized as frequencies and relative frequencies, with continuous variables categorized based on either clinically relevant thresholds or dichotomization at the median. TfR1 immunostain levels (PTP and H-score) were summarized separately for primary tumor tissue, matched benign kidney tissue and metastasis tissue using the mean and standard error (SE) and compared between different tissue types in a pairwise fashion using the Mann–Whitney *U* test. A Spearman correlation coefficient and paired *t*-test were used to compare immunostain H-score levels between matched primary tumors and metastasis tissues, and between matched primary tumors and benign kidney tissues. Associations between TfR1 immunostain scores and patient clinicopathologic variables were evaluated using either the Mann–Whitney *U* test or Kruskal Wallis exact test, as appropriate. For survival analyses, TfR1 H-scores were dichotomized at the median for the ccRCC and non-ccRCC subsets and summarized as low (at or below the median) or high (above the median). Univariable association between low vs. high TfR1 scores and survival outcomes was evaluated using Cox regression models and standard Kaplan–Meier methods, with comparisons made using a log-rank test. Multivariable analyses of high vs. low TfR1 scores and survival outcomes were conducted using Cox regression models, adjusting for age (overall survival only), tumor stage, grade, and size. All models were fit using Firth’s method and hazard ratios (with corresponding 95% CI’s) were obtained from model estimates. Model assumptions were verified graphically using residual plots. All statistical analyses of TfR1 IHC scores were conducted using SAS v9.4 (Cary, NC, USA). Cell line data were summarized using the mean ± SE, with comparisons between groups made using a Student’s two-tailed, unpaired *t*-test with the GraphPad Prism software package v.6.07 (Graphpad Software Inc., San Diego, CA, USA). Cell line gene expression levels determined by RT-PCR were compared by *t*-test comparisons of mean ΔCt values. All statistical analyses were conducted at a significance level of 0.05.

## SUPPLEMENTARY MATERIALS FIGURES AND TABLES



## Abbreviations

ACO1/IRP1: Iron regulatory protein 1
BDI: Bright detail intensity
BMI: Body mass index
ccRCC: Clear cell RCC
chRCC: Chromophobe RCC
FTH1: Ferritin heavy chain
FTL: Ferritin light chain
HAMP: Hepcidi
HIF-α: Hypoxia inducible factor-α
HRCEp: Human renal cortical epithelium
IHC: Immunohistochemistry
IRE: Iron responsive element
IREB2/IRP2: Iron regulatory protein 2
MFI: Mean fluorescence intensity
MSI: Maximum staining intensity
NCI: National cancer institute
non-ccRCC: non-clear cell renal cell carcinoma
PHD: Proline hydroxylase
pRCC: Papillary RCC
PTP: percentage of tissue positivity
RCC: Renal cell carcinoma
RPCI: Roswell Park Cancer Institute
RPTEC: Renal proximal tubule epithelial cell
SE: Standard error
SLC11A2/DMT1: Divalent metal transporter 1
SLC40A1/FPN1: Ferroportin
Tf: Transferrin
TfR2: Transferrin receptor 2
TFRC/TfR1/CD71: Transferrin receptor 1
MA(s): Tissue microarray(s)
VHL: von Hippel Lindau
