# Supplementary material for: Transferrin receptor 1 upregulation in primary tumor and downregulation in benign kidney is associated with progression and mortality in renal cell carcinoma patients
Source: Oncotarget. 2017 Nov 6;8(63):107052–75. doi: 10.18632/oncotarget.22323 (PMC5739796; doi:10.18632/oncotarget.22323)
Supplement: Supplementary file 1 [file oncotarget-08-107052-s001.pdf]

Transferrin receptor 1 upregulation in primary tumor and downregulation in benign kidney is associated with progression and mortality in renal cell carcinoma patients

SUPPLEMENTARY MATERIALS

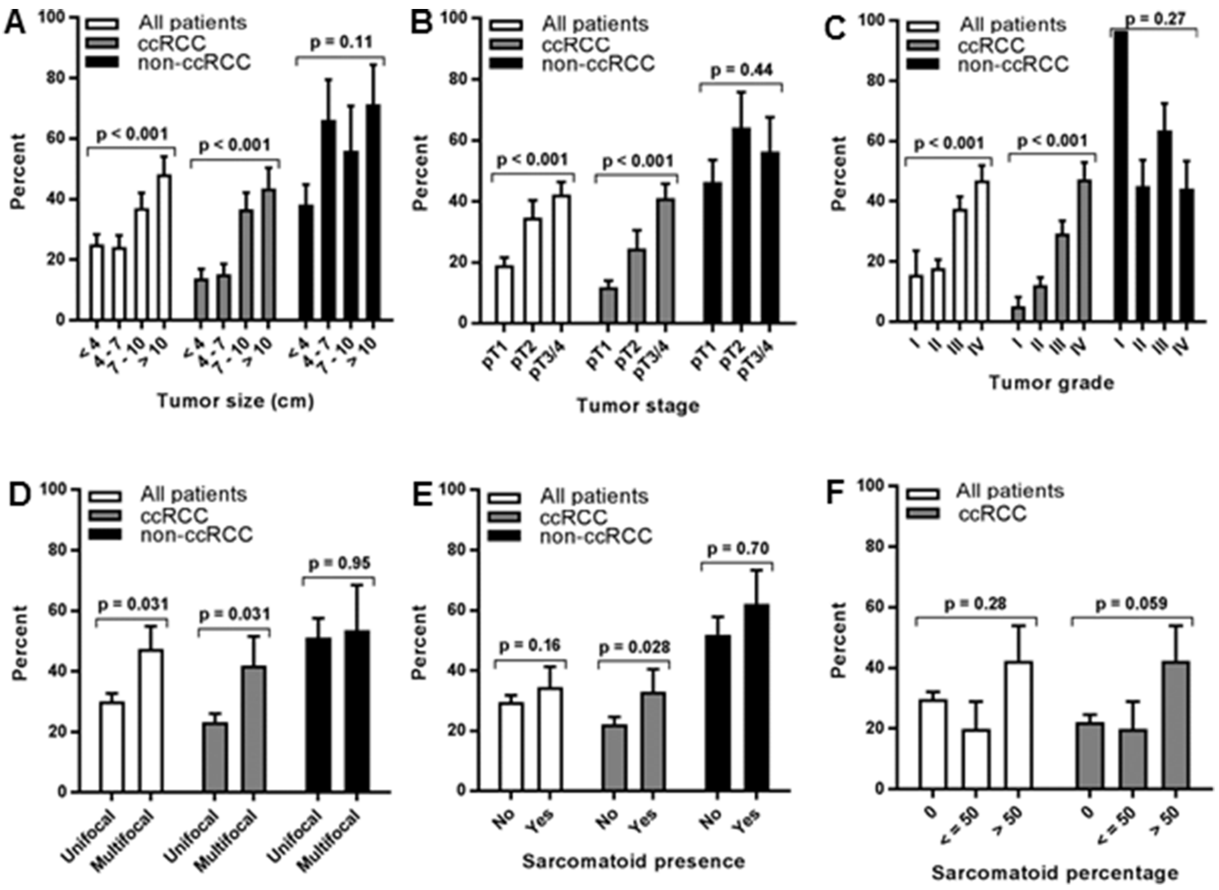

Supplementary Figure 1: Association of primary tumor TfR1 PTP with renal cell tumor pathology. Primary tumor TfR1 level (PTP score) was measured by IHC using renal cell tumor patient TMAs and tested for association with pathologic features of renal cell primary tumors, including **(A)** tumor size (largest diameter), **(B)** tumor stage, **(C)** tumor grade, **(D)** tumor number (focality), **(E)** presence of sarcomatoid dedifferentiation, and **(F)** tumor percentage of sarcomatoid dedifferentiation. Only 1 primary tumor in the non-ccRCC subset was grade I (C), and tumor percentages for two non-ccRCC patients with sarcomatoid dedifferentiation were unknown (F).

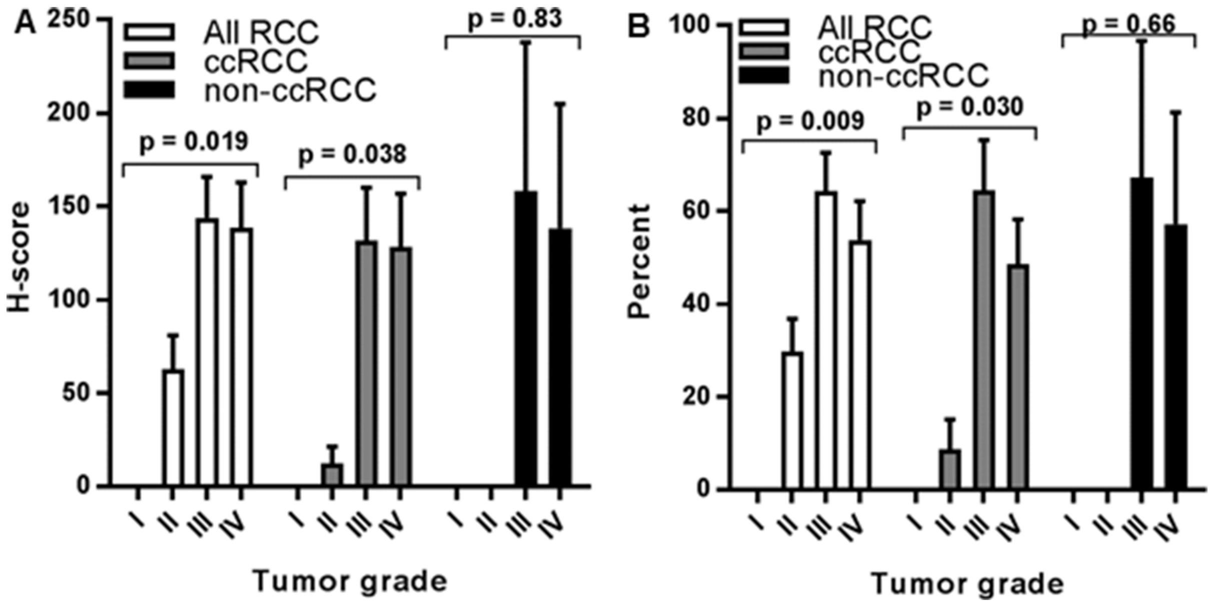

Supplementary Figure 2: Association of metastasis TfR1 levels with RCC primary tumor grade. RCC metastasis TfR1 levels were measured by IHC using renal cell tumor patient TMA and tested for association with pathologic features of RCC primary tumors. Primary tumor grade was significantly associated with metastasis TfR1 levels, including **(A)** mean H-score, and **(B)** mean PTP. No other pathologic variable was significantly associated with metastasis TfR1 levels (data not shown).

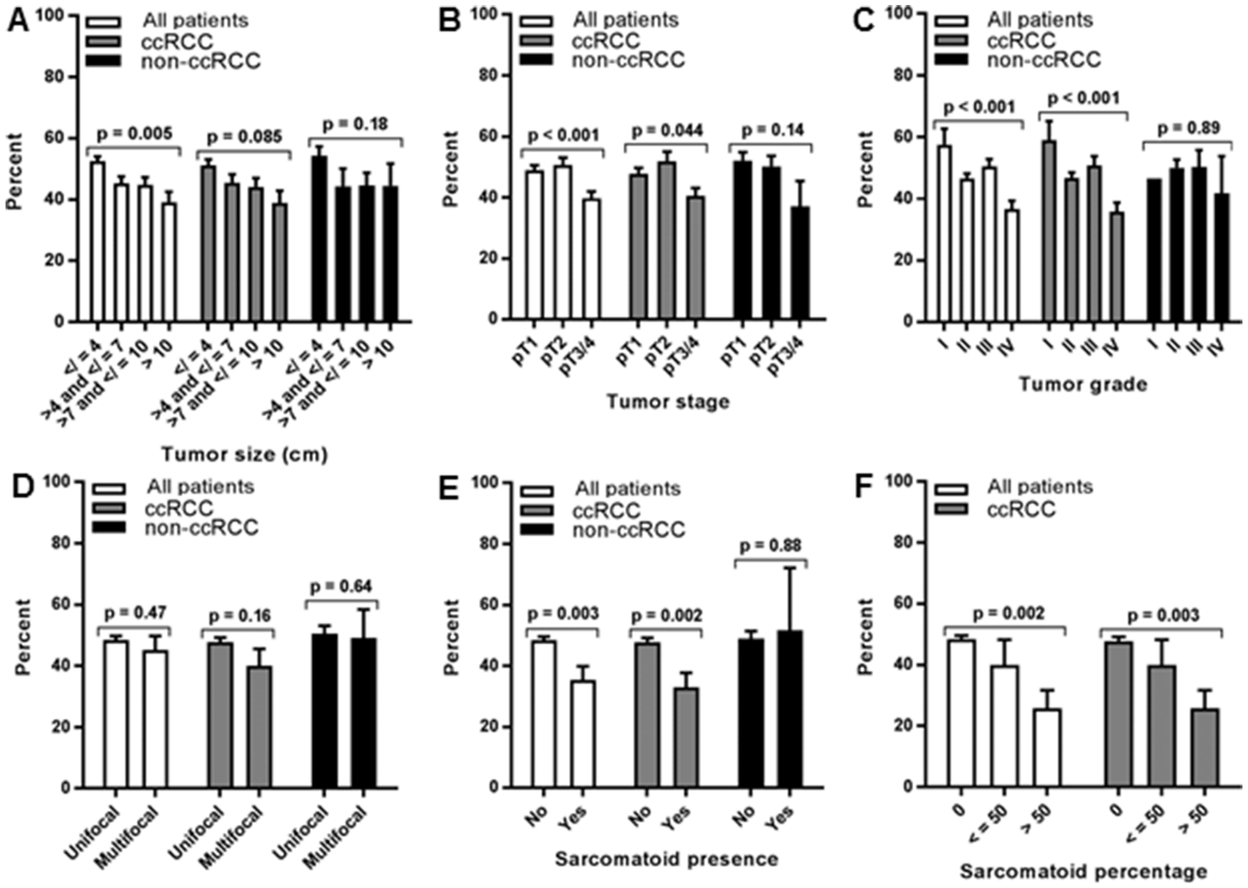

Supplementary Figure 3: Association of benign kidney TfR1 PTP with renal cell tumor pathology. Normal (non-neoplastic) kidney TfR1 level (PTP score) was measured by IHC using renal cell tumor patient TMAs and tested for association with pathologic features of renal cell primary tumors, including **(A)** tumor size (largest diameter), **(B)** tumor stage, **(C)** tumor grade, **(D)** tumor number (focality), **(E)** presence of sarcomatoid dedifferentiation, and **(F)** tumor percentage of sarcomatoid dedifferentiation. Only 1 primary tumor in the non-ccRCC subset was grade I (C), and tumor percentages for two non-ccRCC patients with sarcomatoid dedifferentiation were unknown (F).

Supplementary Table 1: Correlation of TfR1 expression (H-score) between matched renal cell primary tumor and normal kidney tissues

	Spearman correlation		Paired t-test	
	coefficient	p-value	Difference in mean H-score (Primary Tumor – Normal Kidney)	P-value
All patients	-0.10	0.16	-63.1	<0.001
ccRCC	-0.15	0.053	-77.6	<0.001
non-ccRCC	-0.33	0.07	-3.6	0.85

Supplementary Table 2: Correlation of TfR1 expression (H-score) between matched RCC primary tumor and metastatic tissues

	Spearman correlation		Paired t-test	
	coefficient	p-value	Difference in mean H-score (Primary Tumor – Metastasis)	P-value
All RCC	0.47	0.002	-4.6	0.78
ccRCC	0.45	0.016	0.5	0.98

Supplementary Table 3: Association (univariate) between metastasis TfR1 level and RCC patient survival outcomes

TfR1 protein level (PTP or H-score)		Cancer-Specific Mortality		All-Cause Mortality	
		HR (95% CI)	P-value	HR (95% CI)	P-value
ccRCC	PTP	0.78 (0.36, 1.68)	0.51	0.77 (0.37, 1.60)	0.47
	H-score	0.74 (0.34, 1.59)	0.42	0.73 (0.35, 1.52)	0.38
Non-ccRCC	PTP	0.31 (0.05, 1.87)	0.15	0.31 (0.05, 1.87)	0.15
	H-score	0.21 (0.03, 1.68)	0.07	0.21 (0.03, 1.68)	0.07
